# Impact of PSA- versus STN-DBS on effective connectivity in Parkinson’s disease – a 3.0T resting-state fMRI study

**DOI:** 10.1038/s41531-026-01305-y

**Published:** 2026-03-03

**Authors:** Zhengyu Lin, Zhitong Zeng, Chengcheng Duan, Jun Li, Peng Huang, Chencheng Zhang, Qingfang Sun, Bomin Sun, Dianyou Li

**Affiliations:** 1 Department of Neurosurgery, Ruijin Hospital Affiliated to Shanghai Jiao Tong University School of Medicine, Shanghai, China; 2 Center for Functional Neurosurgery, Ruijin Hospital Affiliated to Shanghai Jiao Tong University School of Medicine, Shanghai, China; 3 School of Psychology, Northwest Normal University, Lanzhou, China; 4 Ruijin Hospital LuWan Branch, Clinical Neuroscience Center, Shanghai Jiao Tong University School of Medicine, Shanghai, China

**Keywords:** Neurology, Neuroscience

## Abstract

Subthalamic nucleus deep brain stimulation (STN DBS) is an established treatment for advanced Parkinson’s disease (PD), whereas the posterior subthalamic area (PSA) has been proposed as an alternative target for tremor-dominant cases. However, their underlying therapeutic mechanisms have not been directly compared. Leveraging the single-trajectory dual-target DBS technique, this work utilizes high-field 3.0 T resting-state functional magnetic resonance imaging data and spectral dynamic causal modeling to investigate the differential modulatory effects of PSA and STN stimulation on effective connectivity within both cortico-basal ganglia and cerebello-thalamo-cortical networks. We show that both PSA and STN stimulation suppress cortico-cerebellar connectivity and cortico-subthalamic hyperdirect connectivity, while enhancing STN self-inhibition. Compared with STN stimulation, PSA stimulation provides a greater reduction in cortico-cerebellar coupling but a greater increase in striato-STN connectivity. Moreover, changes in hyperdirect pathway coupling correlate with motor improvement in response to both PSA and STN stimulation. Furthermore, hyperdirect pathway and cerebellar connectivity were significantly associated with motor impairment and resting tremor severity, respectively, regardless of hemisphere or DBS target. Taken together, these findings suggest that PSA and STN stimulation share common network-level mechanisms but differ in their relative modulation of cortico-cerebellar pathway. The present study may offer theoretical guidance for future individualized DBS targeting in treating tremor-dominant PD.

## Introduction

The subthalamic nucleus (STN) deep brain stimulation (DBS) is a well-established neuromodulatory treatment for advanced Parkinson’s disease (PD)^1^. Studies exploring the mechanisms of STN DBS indicate that its neuromodulatory effects extend beyond the target nucleus, engaging multiple levels of the cortico-basal ganglia network. Although it has initially been proposed to mimic the inhibitory effect of ablative lesions in line with firing rate-based models of the basal ganglia^2–4^, electrophysiological^5^ and neuroimaging^6–9^ evidence shows that STN DBS provides broader and multifaceted influences on both local and remote regions from the STN, as well as on the interconnections within them. Beyond the cortico-basal ganglia circuitry, the cerebello-thalamo-cortical network represents another key motor circuit implicated in the pathogenesis of PD, particularly in tremor maintenance^10–13^. The posterior subthalamic area (PSA) is a familiarized target for PD in the lesioning era and is reappraised as DBS target in recent years^14–20^. The robust anti-tremor efficacy of PSA DBS has been consistently documented in various disease conditions, including PD. Furthermore, its beneficial effects on bradykinesia and rigidity have also been recognized^15,18–20^. However, unlike STN, the neuromodulatory effects of PSA DBS remain unexplored. Given the anatomical proximity of these two targets, it is feasible to simultaneously target both PSA and STN using a single trajectory. This targeting approach is particularly effective for severe tremor-dominant PD patients^20,21^. Moreover, from a mechanistic perspective, this configuration offers a unique opportunity to directly compare the neuromodulatory effects of PSA versus STN DBS within the same patient, facilitating a more precise understanding of their distinct therapeutic mechanisms.

When investigating neuromodulatory effects of DBS at rest, blood oxygen level-dependent (BOLD) resting state functional MRI (rs-fMRI) is being increasingly employed^6–9^, offering a favorable balance between spatial and temporal resolution as compared with positron emission tomography/single photon emission computed tomography (PET/SPECT) or electroencephalogram (EEG) modalities. Previous studies have identified STN DBS-related changes in cortico-basal ganglia “effective” connectivity, i.e., changes in the way regions within the cortico-basal ganglia network impact on one another^22,23^. Specifically, using dynamic causal modeling (DCM), a Bayesian approach to estimate the hidden neural states and coupling parameters between and within regions of interest^24–26^, they demonstrated a widespread stimulation-related and clinically-associated changes in the resting-state cortico-basal ganglia network, including decreases in STN afferent and efferent couplings, as well as increases in cortico-striatal, thalamo-cortical and direct pathway coupling^22,23^.

MRI has generally been considered inadvisable for most patients with implanted DBS devices, due to concerns that magnetic fields could interfere with the DBS system circuitry and potentially cause device malfunction. Consequently, earlier imaging studies with DBS-on were almost exclusively limited to 1.5T scanners, older-generation devices, and restricted imaging sequences^6^. With recent technological advances, certain DBS systems have now been labeled for safe 3.0T MRI scanning under DBS-on condition, enabling acquisition of higher-quality images and greatly facilitating research in the related field. Nevertheless, DBS-related neuroimaging studies at higher MR field strengths remain scarce^27^.

In the current study, we modeled both cortico-basal ganglia and cerebello-thalamo-cortical networks using spectral DCM (spDCM) of rs-fMRI data acquired by 3.0T MR in a cohort of PD patients who received bilateral single-trajectory dual-target DBS of the PSA and the STN. We examined the distinct effect of PSA and STN stimulation on the underlying effective connectivity within these networks. As cerebellar efferents ascend and converge near the level of PSA, we hypothesized that PSA DBS may exert a stronger neuromodulatory effect on the cerebello-thalamo-cortical loop compared to STN DBS. Additionally, PSA may also influence the cortico-basal ganglia loop through current spread to the adjacent STN, although such influence is presumed to be less pronounced than with direct STN stimulation. Furthermore, we explored whether clinical benefits after DBS could be explained by changes in effective connectivity within these two networks.

## Results

### Demographics

Fifteen PD patients were enrolled in this study. One patient was scanned at three separate follow-up sessions, and another was scanned twice. These data were treated as independent subjects and thus thirty-six hemispheres were included in the analysis. The mean age at the surgery was 60.9 ± 7.4 years old, the mean disease duration was 10.0 ± 7.4 years, and the median follow-up up to the postoperative scan session was 7.5 (IQR: 5‒11.75) months. Detailed demographics could be found in Table 1. Scanning proceeded with no adverse events related to system impedance or in IPG dysfunction.

**Table 1:** Patient information

ID	Age/Sex	Months since surgery	Baseline MDS UPDRS-III (OFF/ON)	Right electrode				Left electrode			
				contact	amp	pw (μs)	freq (Hz)	contact	amp	pw (μs)	freq (Hz)
1^a^	61/M	9/13/20	52/21	c + 1−	2.5	60	145	c + 9−10−	2.6	50	145
2	69/M	19	50/23	c + 1−	2.9	50	160	c + 10−	3.0	50	160
3	61/M	6	37/13	c + 2−	3.7	40	160	c + 10−	3.5	50	160
4^a^	66/F	5/16	46/17	c + 1−	3.5	50	160	c + 9−	2.9	50	160
5	65/M	5	40/15	c + 0−/c + 1−	1.5/2.5	60	125	c + 8−/c + 9−	1.5/2.6	60	125
6	67/M	5	53/27	c + 2−	1.6	50	60	c + 9−10−	2.6	50	120
7	59/M	5	69/25	c + 0−/c + 1−	1.75/2.6	50	60/120	c + 9−/c + 10−	2.2/3.25	50/70	60/120
8	54/M	5	64/36	c + 2−	2.9	40	90	c + 10−	3.0	40	90
9	51/M	4	53/19	c + 1−	1.85	60	125	c + 10−	2.65	50	125
10	66/F	8	67/26	c + 1b-1c−	4.1	60	150	c + 9−/c + 11−	3.9/2.7	50/60	75/150
11	67/M	12	56/26	c + 1−2−	2.5	50	160	c + 9−	2.15	50	160
12	61/F	11	53/37	c + 1−/c + 3−	2.75/3.5	50	125	9 + 10−/10 + 11−	3.5/3.5	70/90	125
13	64/M	7	52/17	c + 1−/c + 2-	2.5/2.75	50	130	c + 9−10-	3.05	40	130
14	38/F	7	73/32	c + 1−	3.25	50	160	c + 8−	2.9	50	160
15	60/M	8	65/18	c + 0−1−/c + 2−	3.25/2.25	50	160	c + 8−/11 + 10−	3.25/3.3	50	160

*amp* amplitudes, *pw* pulse width, *freq* frequency.

^a^Participants received two or three sessions of scans after surgery and were treated as independent samples in the analysis.

### Clinical benefits of DBS

Compared to preoperative off-medication baseline, both PSA and STN DBS significantly improved the total MDS UPDRS-III score (OFF: 55.0 ± 9.8; PSA: 29.2 ± 8.0; STN: 27.7 ± 12.3; ${\eta }_{p}^{2}$ = 0.833), as well as sub-scores for resting tremor (OFF: 6.8 ± 4.1; PSA: 1.4 ± 2.3; STN: 1.1 ± 2.1; ${\eta }_{p}^{2}$ = 0.820), bradykinesia (OFF: 25.1 ± 6.3; PSA: 15.8 ± 4.7; STN: 15.2 ± 6.4; ${\eta }_{p}^{2}$ = 0.647), rigidity (OFF: 11.5 ± 2.5; PSA: 4.8 ± 2.0; STN: 4.7 ± 2.8; ${\eta }_{p}^{2}$ = 0.561), and axial symptoms (OFF: 7.8 ± 3.9; PSA: 5.2 ± 2.9; STN: 4.7 ± 3.0; ${\eta }_{p}^{2}$ = 0.559) (all *p* < 0.001). No significant difference in motor improvement was observed between PSA and STN stimulation.

### Accuracy of spDCM model estimation

The estimation of spDCM models for individual participants for each session was good to excellent: the average percentage variance explained using spDCM estimation was 93.3% (range: 85.5–95.3%).

### spDCM with PEB

**Fig. 1:**
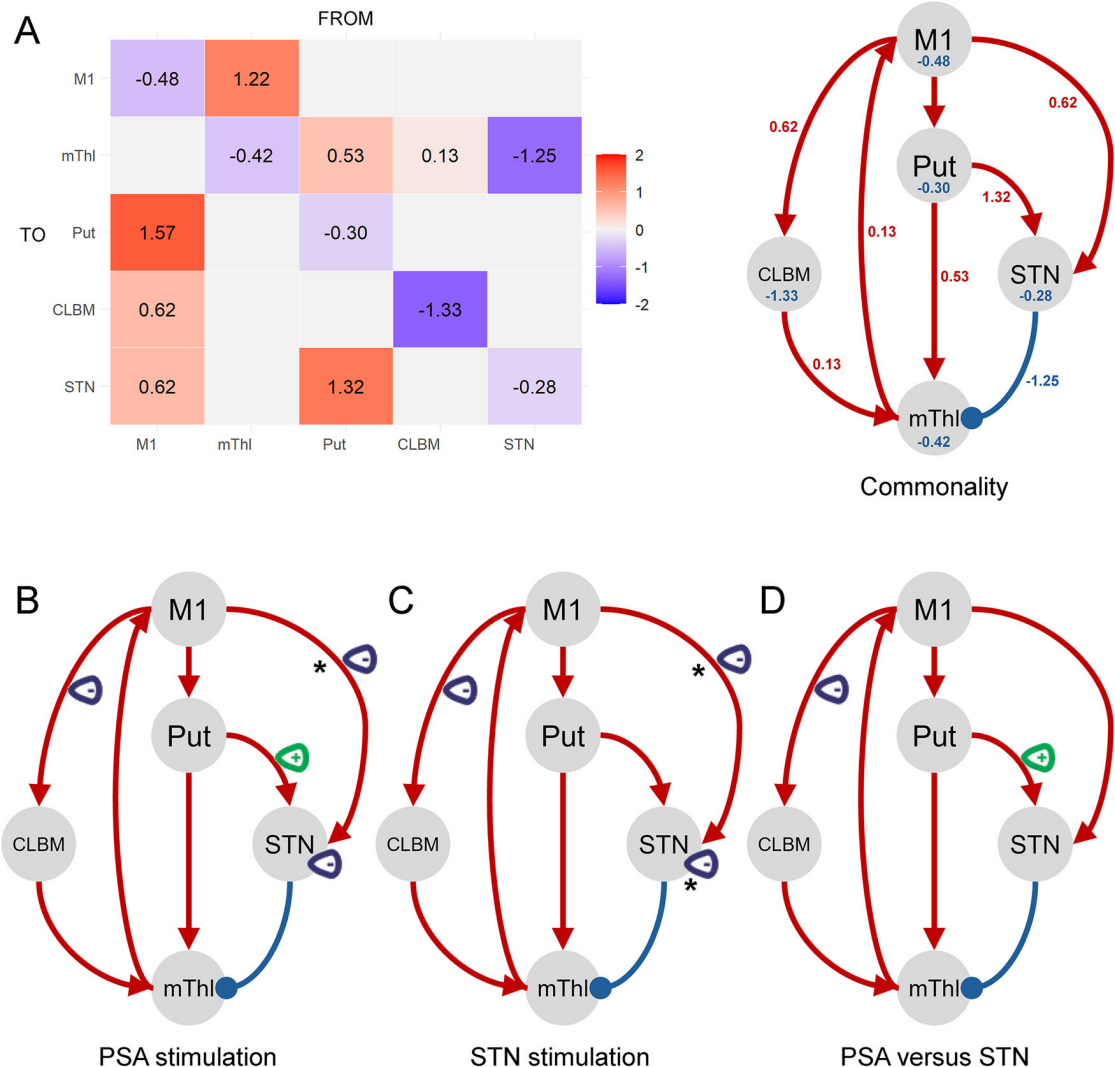
Baseline and stimulation-specific connectivity changes. The shared connection strengths matrix across participants and conditions were shown in (**A**). Coupling parameter changes in PSA (**B**), STN (**C**), and between PSA and STN (**D**) stimulation condition with posterior probability (Pp) > 0.75, indicating positive evidence (Kass and Raftery^38^), were shown. *indicated the connectivity associated with clinical motor outcome (*p* < 0.05).

Figure 1A showed the shared connection strengths quantified by posterior estimate (Ep) in Hz across participants and conditions. Considering the nature of the DBS modulatory effect, our analysis focused on changes in extrinsic coupling and intrinsic STN connectivity. PEB-of-PEBs analysis revealed a decrease in M1 → CLBM connectivity of −0.26 Hz (Pp = 0.98) and M1 → STN connectivity of −0.11 Hz (Pp = 0.81), along with an increase in Put→STN connectivity of +0.10 Hz (Pp = 0.78). In addition, PSA stimulation increased STN self-inhibition by +9.30% (Pp = 0.77). Similar effects were observed during STN stimulation, including reduction in M1 → CLBM (−0.18 Hz, Pp = 0.93), M1 → STN connectivity (−0.16 Hz, Pp = 0.90), and a +18.6% increase in STN self-inhibition (Pp = 0.92). Direct comparison revealed that PSA stimulation weakened M1 → CLBM connectivity further (ΔEp = −0.11 Hz, Pp = 0.82) but enhanced Put→STN (ΔEp = 0.15 Hz, Pp = 0.90) connectivity relative to STN stimulation (Fig. 1B–D). Similarly, the linear mixed-effects model analysis revealed that the M1 → CLBM connectivity [F (2, 36.54) = 4.765; ${\eta }_{p}^{2}$ = 0.207; *p* = 0.014], M1 → STN connectivity [F (2, 34.82) = 3.444; ${\eta }_{p}^{2}$ = 0.165; *p* = 0.032], and Put→STN connectivity [F (2, 36.72) = 3.296; ${\eta }_{p}^{2}$ = 0.152; *p* = 0.048] were affected by stimulation condition. The Post-hoc comparisons indicated a significant decrease in M1 → CLBM connectivity (adjusted *p* = 0.012) under PSA stimulation compared to off-stimulation, a significant decrease in M1 → STN connectivity (adjusted *p* = 0.035) under STN stimulation compared to off-stimulation, as well as a trend of increase in Put→STN connectivity (adjusted *p* = 0.056) under PSA stimulation compared to STN stimulation (Supplementary Fig. 2).

### Correlation analysis

**Fig. 2:**
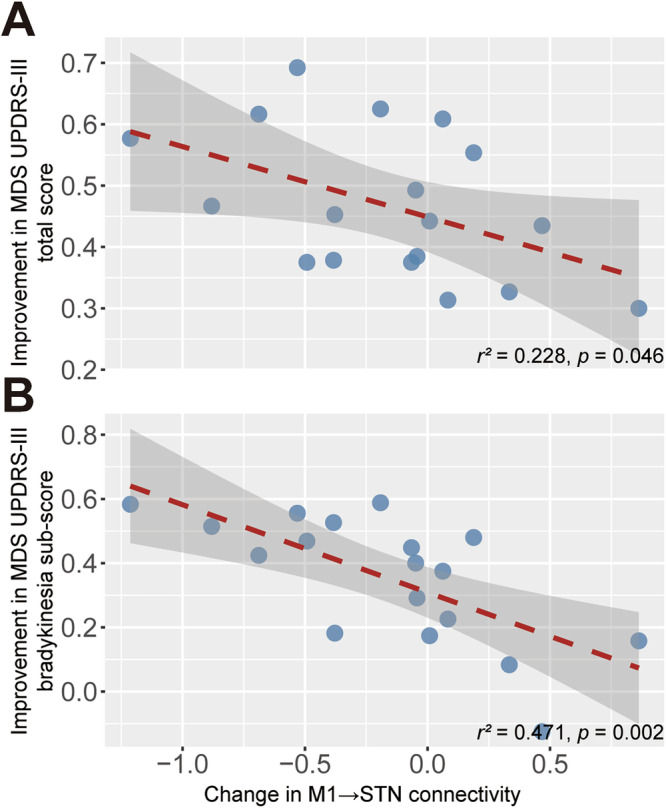
Correlation analysis under PSA stimulation. **A** Change in M1→STN coupling versus improvement inMDS-UPDRS III total score; (**B**) change in M1→STN coupling versus improvement in MDS-UPDRS III bradykinesia sub-score. Red dashed line represents the linear regression line, and the gray area represents the 95% confidence interval. The square of Pearson’s r and *p*-values are reported.

**Fig. 3:**
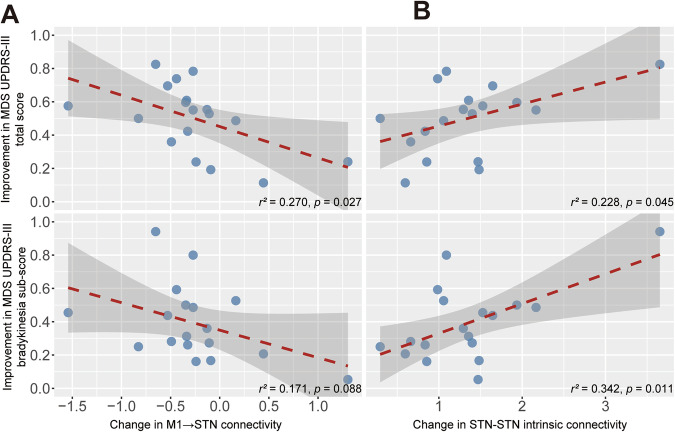
Correlation analysis under STN stimulation. **A** Change in M1→STN coupling versus improvement in MDS-UPDRS III total score and bradykinesia sub-score; (**B**) change in STN–STN intrinsic connectivity versus improvement in MDS-UPDRS III total score and bradykinesia sub-score. Red dashed line represents the linear regression line, and the gray area represents the 95% confi dence interval. The square of Pearson’s r and *p*-values are reported.

Correlation analysis showed that the change in total score (*r*^2^ = 0.228; *p* = 0.046) and bradykinesia sub-score (*r*^2^ = 0.471; *p* = 0.002) of MDS UPDRS-III was negatively correlated with change in M1 → STN connectivity under PSA stimulation (Fig. 2). In case of STN stimulation, improvements in total score of MDS UPDRS-III correlated negatively with change in M1 → STN connectivity (*r*^2^ = 0.270; *p* = 0.027) and positively with change in STN-STN intrinsic connectivity (*r*^2^ = 0.228; *p* = 0.045). Improvements in bradykinesia sub-score of MDS UPDRS-III showed a trend towards negative correlation with change in M1 → STN connectivity (*r*^2^ = 0.171; *p* = 0.088) and correlated positively with change in STN-STN intrinsic connectivity with statistical significance (*r*^2^ = 0.342; *p* = 0.011). (Fig. 3). We also conducted exploratory analyses examining correlations between lateralized motor scores and the corresponding hemisphere-specific DCM coupling parameters across stimulation conditions. The lateralized total and bradykinesia sub-score of MDS UPDRS-III exhibited weak but significant positive correlations with M1 → STN coupling parameter (total: rho = 0.225, *p* = 0.008; bradykinesia: rho = 0.217, *p* = 0.005). Additionally, the resting tremor sub-score showed weak but significant positive associations with both cerebellar afferent (rho = 0.175, *p* = 0.016) and efferent (rho = 0.300, *p* = 0.002) coupling parameters (Supplementary Fig. 2).

## Discussion

This study is the first to identify the modulatory effects of PSA DBS on effective connectivity of both cortico-basal ganglia and cerebello-thalamo-cortical networks, and to compare effects of PSA versus STN DBS on effective connectivity of these two networks in PD. We demonstrated the following: (i) decreased cerebellar afferent (M1 → CLBM) and M1 → STN hyperdirect pathway connectivity, as well as increased self-inhibition of STN connectivity under both PSA and STN stimulation; (ii) further decreased cerebellar afferent (M1 → CLBM) connectivity, but increased Put→STN indirect pathway connectivity under PSA stimulation compared to STN stimulation; (iii) positive correlation between DBS-related clinical improvements and changes in hyperdirect pathway coupling as well as in STN intrinsic coupling.

Observations in this study aligned in part with those reported in earlier work. Specifically, within the cortical basal ganglia network, reductions in M1 → STN hyperdirect pathway alongside increased STN self-inhibition were consistent with prior evidence that therapeutic STN DBS suppressed M1 → STN coupling^22,28^ and hyperexcitability of STN^5,29,30^. Not surprisingly, given the close anatomical adjacency and since we used adjacent active contacts for PSA and STN stimulation scanning sessions, the changes in effective connectivity induced by PSA stimulation were similar to those observed under STN stimulation. Correlation analysis revealed a positive association between the strength of the hemispheric cortico-STN coupling and the severity of contralateral motor impairment across stimulation conditions. Furthermore, both the magnitude of STN DBS-induced reduction in cortico-STN coupling and the strength of increased STN self-inhibition correlated with motor improvement. This is in line with electrophysiological findings showing that effective STN stimulation selectively attenuates hyperdirect-driven beta oscillations and that the degree of suppression in STN oscillations correlates with clinical benefit^31^. Significant correlation between clinical benefit and hyperdirect pathway coupling strength was also found under PSA stimulation.

For cerebello-thalamo-cortical network modeling, both cerebellar afferent (i.e., M1 → CLBM) and efferent (CLBM→mThl) coupling parameters were positively associated with the severity of the resting tremor across stimulation conditions. This aligns with the “dimmer-switch” theory, which posits that the basal ganglia act as a “switch” to trigger Parkinsonian tremor onset, while the cerebello‑thalamo‑cortical circuit functions as a “dimmer” to maintain and modulate its amplitude^12,13^. We initially anticipated that PSA and STN stimulation would modulate cerebellar efferents, consistent with the anatomical basis of the dentato‑rubro‑thalamic tract (DRTT) who passes through the vicinity of the STN. However, our findings suggest that both PSA and STN stimulation primarily suppressed afferent inputs from cortex to CLBM, rather than modulating its efferent projection towards the thalamus, with PSA exerting a stronger inhibitory effect than STN stimulation. This pattern can be explained by the notion that the consequences of antidromic stimulation are ultimately expressed orthodromically in cortical efferent (and afferent) pathways connected to the basal ganglia or the cerebellum^5^. Furthermore, the observation that PSA stimulation results in a greater reduction in coupling parameters of cortico-cerebellar afferent without a significantly superior tremor reduction may suggest a ‘diminishing returns’ relationship. In other words, while PSA is more potent in decoupling the circuit, the clinical tremor suppression may have already reached a functional ceiling with STN stimulation, such that the additional physiological gain of PSA does not manifest as a statistically distinct clinical advantage in this sample size.

Our results provide a mechanistic rationale for personal target selection in PD. PSA is a white-matter-dense region—mainly consisting of the caudal zona incerta and the prelemniscal radiation—located immediately posterior, medial, and superior to STN. It serves as a critical junction where both cerebellar and pallidal fibers converge before entering the thalamus^14^. In contrast, STN is a key hub within the basal ganglia loop, receiving dense cortical inputs and subcortical projections predominantly from the output nuclei of the basal ganglia. A recent computational modeling study showed that PSA-DBS could simultaneously recruit both the hyperdirect and cerebellothalamic pathways while avoiding excessive capsular activation. In contrast, STN-DBS resulted in higher levels of recruitment of the hyperdirect pathway only but was limited by capsular activation at higher stimulus amplitudes^32^. Aligned with this prior study, we observed that PSA stimulation exerted a more potent inhibitory effect on cerebellar afferent connectivity along with hyperdirect pathway involvement. This suggests that PSA-DBS may be particularly advantageous for PD patients with severe tremor, as it may provide superior decoupling of the cerebello-thalamo-cortical “dimmer” circuit that maintains tremor oscillations^12,13^. Instead, the robust modulation of hyperdirect connectivity under STN stimulation supports STN as a primary target for broad motor symptom relief, including rigidity and bradykinesia. In addition, these findings support an optimization of STN targeting in PD. Through the strategic placement of directional lead along the posteromedial border of the STN, clinicians can steer the current toward the dorsolateral STN “sweet spot” and the PSA simultaneously. Crucially, this dual-targeting method will allow personalized DBS management for TD-PD patients with severe tremor, enabling clinicians to adjust the percentage of current delivered to STN versus PSA to more effectively suppress tremor, rigidity, and bradykinesia while minimizing off-target effects. These insights shift the perspective of DBS from “one-size-fits-all” to a circuit-based approach, where the targeting strategy and contact choice can be tailored to the patient’s specific network dysfunction profile.

However, our work did not replicate several modulatory effects of STN DBS previously reported^22,23^, including changes in cortico-striatal, striato-thalamic, and STN-thalamic couplings. These discrepancies likely reflect differences in rs-fMRI acquisition protocol, DCM modeling and inversion approach, and analytical framework. Specifically, previous work implemented a two-state stochastic DCM with STN treated as “hidden” node due to its small size, the relatively low spatial resolution of the rs-fMRI acquisition protocol, and artefacts introduced by DBS electrodes. They also concatenated the DBS-ON and DBS-OFF sessions into one single continuous time-series, treating stimulation state as “condition” in the GLM design matrix. This allowed the modulatory effects of stimulation to be modeled in the B-matrix of DCM. The decision to concatenate DBS‑ON and DBS‑OFF sessions into a single time‑series may be appropriate when all scans are acquired during the same clinical visit, ensuring consistency in temporal context and minimizing between-session variability. However, in our study, rs‑fMRI data were collected at different time points, and this temporal dispersion rendered the concatenated GLM/DCM approach less suitable. Indeed, stochastic DCM on our concatenated data only resulted in a modest accuracy of DCM model estimation (data not shown). Therefore, we modeled each session separately using spDCM with a hierarchical PEB-of-PEBs framework. In spDCM, the model estimates time-invariant effective connectivity parameters by fitting the cross‑spectral density of observed BOLD signals across regions, rather than modeling the original time series directly. As our study leveraged a higher spatial resolution of 3.0T rs-fMRI acquisition protocol compared to that used in the previous work, it reduces partial volume contamination and improves our ability to sample the “true” STN signal.

Another factor that may contribute to discrepancy is that previous studies leveraged a task-based fMRI protocol to localize individualized M1 coordinates for each participant. They then used psychophysiological interaction analysis to identify voxels within putamen and thalamus masks whose activity was associated with both M1 activation and DBS stimulation state, thereby facilitating more precise model inversion and modulatory parameter estimation. In contrast, we relied on literature-derived MNI M1 coordinates associated with hand function. The use of standardized rather than individualized functional locus represents one limitation of our work.

Similar to previous work, our model made several simplifying assumptions, including the independence of the right and left hemisphere cortico-basal ganglia and cerebello-thalamo-cortical circuits, the absence of pallidal dynamics by simplifying the basal ganglia motor loop, and the sparsity of connections amongst the nodes. DCM does not necessarily quantify monosynaptic coupling. Thus, not all intermediate nodes are required to estimate effective connectivity between any two nodes. In addition, we used a relatively loose posterior probability of 0.75 and further research is needed to validate our findings.

Finally, whereas treating multiple postoperative scans as independent samples could bias interpretations, we mitigated this concern by implementing linear mixed-effects model alongside the PEB-of-PEBs framework. Although findings are reported at an uncorrected threshold—a noted limitation—the emphasis on effect size provides a critical metric for evaluating robustness. Specifically, the observed large effect sizes suggest that the stimulation-induced changes reflect non-negligible biological effects rather than stochastic noise, providing a reliable basis for future validation in larger cohorts. Nevertheless, the small sample size remains a major limitation of this study, and these results should be interpreted as preliminary. Beyond these acute dynamics, the long-term impact of chronic stimulation of PSA- versus STN-DBS on effective connectivity remains unaddressed. Elucidating such longitudinal effects on motor, non-motor, and quality-of-life outcomes represents another trajectory for subsequent research.

To conclude, this study is the first to report the differential neuromodulatory effects of PSA and STN DBS on effective connectivity within both the cortico-basal ganglia and cerebello-thalamo-cortical circuits in PD. PSA DBS appeared to exert a greater influence on the cerebellar connectivity, which is associated with the clinical severity of Parkinsonian tremor. Moreover, our findings further highlight the notion that modulation on the hyperdirect pathway and STN self-inhibition may represent core mechanisms underlying the therapeutic effects of DBS. These findings may offer theoretical guidance for future individualized DBS targeting in treating tremor-dominant PD.

## Methods

### Participants

The study was performed in accordance with the Declaration of Helsinki, and the protocol was approved by the ethics committee of Ruijin Hospital Affiliated to Shanghai Jiao Tong University School of Medicine, Shanghai, China (approval numbers: 2023-212; 2024-429). Fifteen participants were prospectively enrolled. Inclusion criteria were as follows: (i) a clinical diagnosis of PD according to the MDS Clinical diagnostic criteria as determined by a movement disorder specialist; (ii) tremor-dominant subtype^33^; (iii) underwent single-trajectory PSA-STN dual-target DBS surgery^20,21^ and with stabilized stimulation parameters; (iv) had 3.0T MR-compatible DBS system implantation allowing for scanning during on-stimulation condition; (v) could tolerate lying flat with minimal head tremor while being off-medication condition before surgery; and (vi) no evidence of cognitive deficit, defined as Mini-Mental State Examination (MMSE) score of <24. Written informed consent for participation was obtained from each participant before enrollment.

### rs-fMRI data acquisition

Medication was withdrawn for at least 12 h (overnight) prior to scanning. Participants underwent one rs-fMRI scan before surgery, and subsequent two postoperative scans under PSA and STN stimulation, respectively. In accordance with the manufacturer’s requirements, participants implanted with a Medtronic DBS system using ring-mode electrodes (first 7 cases; electrode model 3389, IPG Percept^TM^ PC, Medtronic) underwent bipolar stimulation during scanning. Subjects implanted with a PINS DBS system (cases 11‒15; electrode model L301CS, IPG G106R or G106RS, PINS) underwent monopolar stimulation. For subjects implanted with a Medtronic DBS system using directional electrodes (cases 8–10; electrode model B33005, IPG Percept™ PC, Medtronic), as bipolar stimulation was not available in directional mode, and considering published safety data^34^, monopolar stimulation was used in this subgroup. We closely monitored the specific absorption rate during scanning, and no adverse events related to impedance changes or DBS system malfunction were documented at any time after each scan. The rs-fMRI images (3.0-T UIH uMR890, United Imaging, China) were acquired with the following parameters: TR = 0.7 s, TE = 30 ms, multiband acceleration factor = 7, voxel size = 2.5 mm isotropic, no slice gap. In addition to two fieldmap scans, an anatomical T_1_-weighted MR-RAGE structural scan was also acquired. The order of postoperative scans was counterbalanced. Each postoperative scan started at least 30 min after switching the stimulating parameter. After each scan, participants underwent a videotaped MDS‑UPDRS‑III evaluation, and recordings were subsequently rated by an experienced, blinded rater. Both the rater and the participant were unaware of the stimulation condition during assessment.

### Stimulation settings

The respective stimulation parameters for PSA and STN were configured to match each participant’s chronic and stable clinical settings. Based on fused postoperative CT and preoperative MRI images, stimulation contacts located within the PSA and STN were selected accordingly. For ring-mode electrodes (Medtronic 3389 or PINS L301CS), the PSA and STN contacts were typically adjacent. In patients treated with directional DBS, electrodes (model B33005, Medtronic) were deliberately positioned at the border of the STN to allow selective steering of directional contacts to both the PSA and STN. The selection of directional contacts for both PSA- and STN-oriented stimulation was limited to a single segmented contact per direction. To this end, the position of the directional electrode in relation to the subthalamic area was verified using the fused image of postoperative CT and preoperative MR (Supplementary Fig. 1). The orientation of the DBS lead marker was confirmed on anteroposterior and lateral skull X-ray acquired one month after surgery. In addition, the directional contact oriented toward the PSA and STN was further verified based on the monopolar review. Stimulation frequency and pulse width were typically set at 130 Hz and 60 µs, respectively, but could be finetuned according to the individual stable clinical setting. Stimulation amplitude was increased stepwise and was set at the maximum tolerable level without stimulation-induced side effects (e.g., capsular reactions, dysarthria, stimulation-induced dyskinesia, paresthesia, etc.).

### Preprocessing

**Fig. 4:**
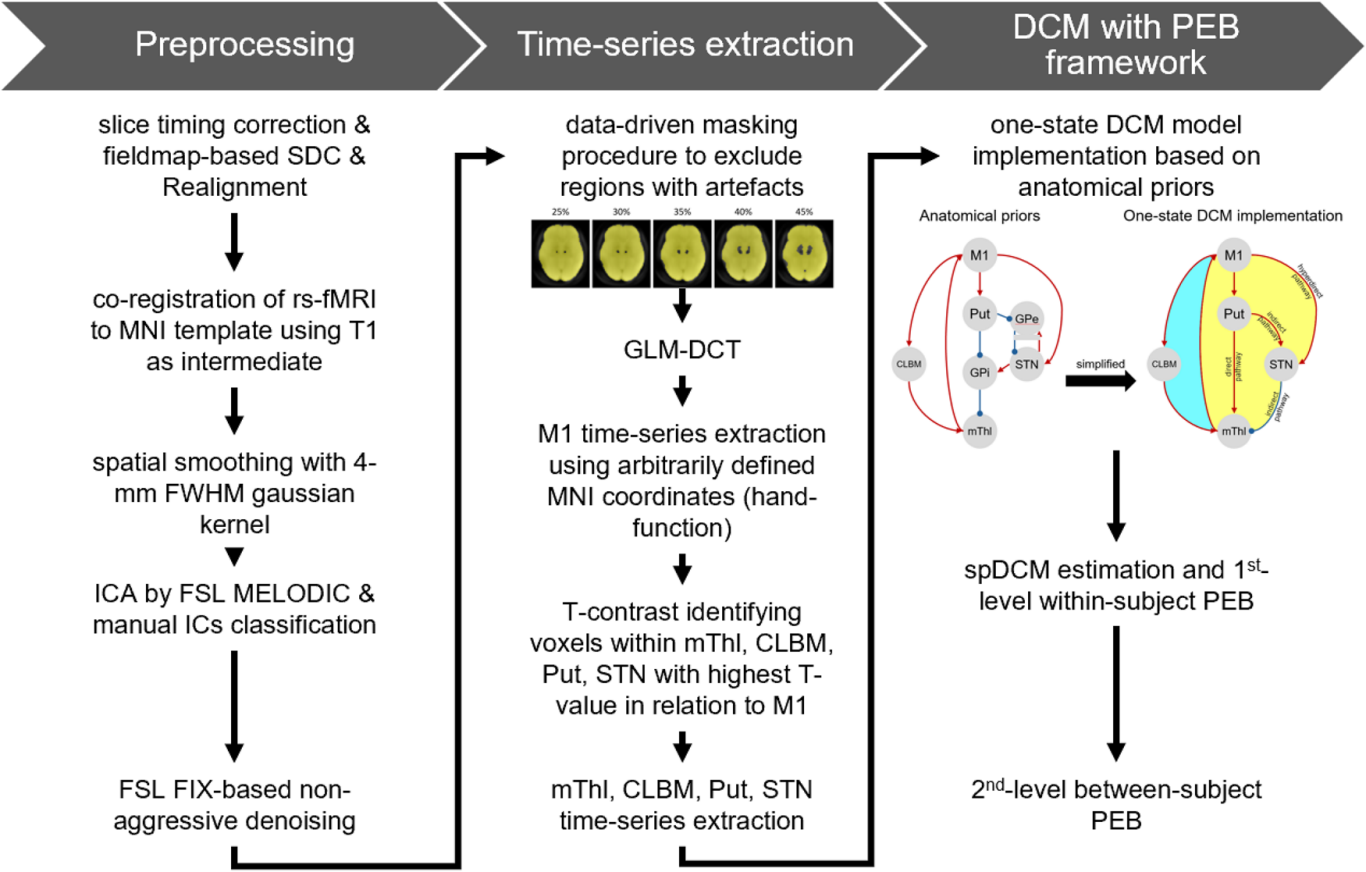
The pipeline of rs-fMRI data preprocessing and dynamic causal modeling (DCM) analysis. Data analysis included three main steps, including preprocessing, region of interest time-series extraction, and DCM with two-level parametric empirical Bayesian framework (PEB).

The pipeline of rs-fMRI data preprocessing was illustrated in Fig. 4. All preprocessing steps were performed using FSL (the FMRIB Software Library, v6.0.7.17), and included slice timing correction, fieldmap-based susceptibility distortion correction, motion correction, co-registration to MNI standard space using T_1_-weighted images as intermediate, 200-s high-pass filtering, spatial smoothing with 4-mm FWHM Gaussian kernel, and FSL FIX-based non-aggressive denoising. Preprocessed, denoised rs-fMRI data were then analyzed using a general linear model (GLM) to extract time-series data.

### General linear model of resting-state dynamics

The post-preprocessing analysis was performed in SPM12 (https://www.fil.ion.ucl.ac.uk/spm/software/spm12/, r7771). The rs-fMRI data of each session for each participant were analyzed separately. Data was modeled using a GLM with a discrete cosine basis set (GLM-DCT) and regressors encoding motion outliers. The motion outlier was defined as the volume with framewise displacement >0.3 mm or standardized DVARS > 1.5. A data-driven mask procedure developed by our group^9^ was used to create a brain mask with an intensity cutoff threshold of 25% to exclude voxels exhibiting severe magnetic susceptibility artifacts caused by DBS apparatus. This specific masking pipeline was also successfully applied to STN BOLD time-series in our previous work^7^. The regional BOLD signal was summarized with the principal eigenvariate of voxels within 4 mm of the arbitrarily defined MNI coordinate of M1 representing the hand functions (left hemisphere: *x* = −37, *y* = −21, *z* = 58; right hemisphere: *x* = 38, *y* = −17, *z* = 58)^35^. Subsequently, a T-contrast was computed with respect to M1 activity to identify voxels with highest *T*-value within thalamus, putamen, STN, and cerebellum. The BOLD signal was extracted from a sphere of 4-mm radius centered on the peak *T*-value within each mask, producing five volumes of interest per hemisphere and subject (M1, putamen, thalamus, STN, and contralateral cerebellum).

### Spectral DCM with parametric empirical bayes

DCM is a Bayesian framework to model effective connectivity, representing the causal, directed influences between and within volumes of interest^36^. The traditional DCM treats the brain as a ‘deterministic’ system without modeling “endogenous” fluctuations intrinsic to brain regions. The stochastic DCM, instead, extends the original deterministic DCM by incorporating spontaneous noisy fluctuations into the state equations, allowing one to model dynamics without experimental inputs, for example, in the “resting state”^24^. Building upon this framework, spectral DCM (spDCM) has been developed to analyze the cross-spectral density of these fluctuations rather than raw time-series, offering a more robust and computationally efficient approach for estimating effective connectivity. In the present study, we therefore utilized one-state (generic) spDCM and the model was specified based on anatomical priors adapted from Kahan J. et al.^22^. The pallidal dynamics were omitted from the current DCM framework primarily to maintain methodological consistency with previous benchmark studies on STN-DBS effective connectivity^22^, thereby ensuring that our findings regarding PSA-DBS could be directly contextualized within the existing literature. Specifically, within the cortico-basal ganglia network, the direct pathway was defined as the striato-thalamic excitatory connection, and the indirect pathway consisted of both the striato-STN and STN-thalamic connections. The hyperdirect pathway was defined as the M1-STN connections. In the cerebello-thalamo-cortical network, the ascending pathway was represented by the cerebello-thalamo-M1 connection, whereas the descending pathway was defined as the connection from M1 to the cerebellum.

A separate model was constructed for each session and hemisphere of each participant, and was inverted to provide estimates of coupling parameters, i.e., the intrinsic and extrinsic effective connectivity strengths. Subsequently, we applied hierarchical parametric empirical bayes (PEB) to estimate effective connectivity at both within-subject and between-subject level. This PEB-of-PEBs framework leverages uncertainty in individual estimates to obtain robust group inferences about both common (“baseline”) and condition-specific connectivity effects, while preserving parameter uncertainty for hypothesis testing and behavioral correlation^37^. The hemispheric laterality did not have a significant impact on coupling parameters so that both hemispheres were averaged in the statistical analysis (data not shown). Specifically, In the second-level PEB analysis, a GLM was constructed to identify the commonalities in effective connectivity across all experimental conditions (OFF + PSA + STN) and the condition-specific differences (PSA vs OFF; STN vs OFF, and PSA vs STN). The age, sex, and follow-up duration were incorporated as covariates of no interest. The posterior probability (Pp) quantifies the confidence in the presence and directionality of an estimated coupling parameter, given the data and prior assumptions. A Pp value > 0.75 (defined as “positive evidence” for a nonspurious effect^38^) was applied.

### Statistical analysis

Apart from the PEB-of-PEBs framework mentioned above, a linear mixed-effects model was also constructed with stimulation condition as fixed factor and subject ID as random factor for total and sub-scores of the MDS UPDRS-III as well as coupling parameters. We also incorporated age, gender, and follow-up duration as fixed-effect covariates to statistically control for their potential impact. The effect size was quantified using partial eta squared (${\eta }_{p}^{2}$). Given the exploratory nature of this study and the relatively small sample size, we reported raw p-values to avoid Type II errors (missing potential effects). To ensure the robustness of our findings, we prioritized coupling parameters that achieved both *p* < 0.05 and a large effective size (${\eta }_{p}^{2}$ > 0.14)^39^. In the event of a significant main effect, post-hoc pairwise comparisons were performed (Tukey’s HSD corrected). We also performed a correlation analysis between coupling parameters that differed across stimulation conditions and motor outcome measures. Statistical analysis was conducted using R software (version 4.5.1).

## Supplementary information

Supplementary Figure

## Data Availability

The datasets generated and analyzed during the current study are not publicly available due to ethical restrictions, but are available from the corresponding author on reasonable request within the limits of participant’s consent.
