# Supplementary material for: Impact of PSA- versus STN-DBS on effective connectivity in Parkinson’s disease – a 3.0T resting-state fMRI study
Source: NPJ Parkinsons Dis. 2026 Mar 3;12:92. doi: 10.1038/s41531-026-01305-y (PMC13065830; doi:10.1038/s41531-026-01305-y)
Supplement: Supplementary file 1 — Supplementary Figure [file 41531_2026_1305_MOESM1_ESM.pdf]

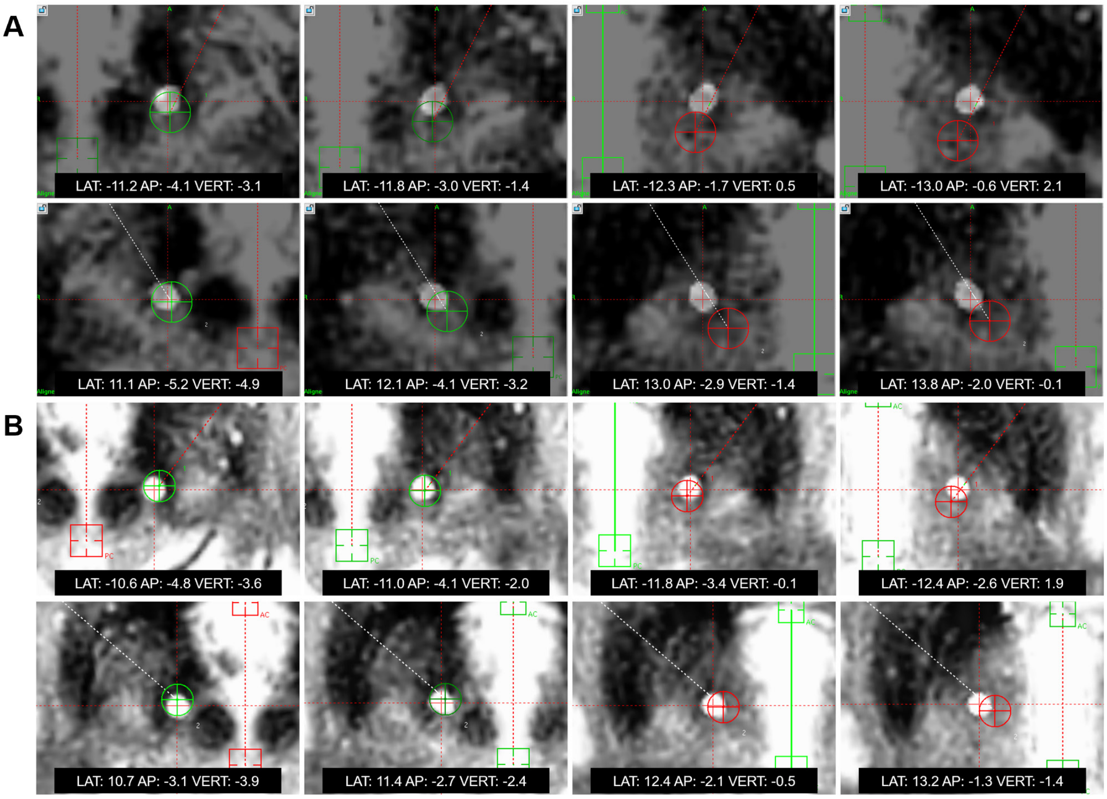

Supplementary Figure 1. The fused images of postoperative CT and preoperative MR of representative cases with ring-mode (A) and directional-mode electrodes (B).

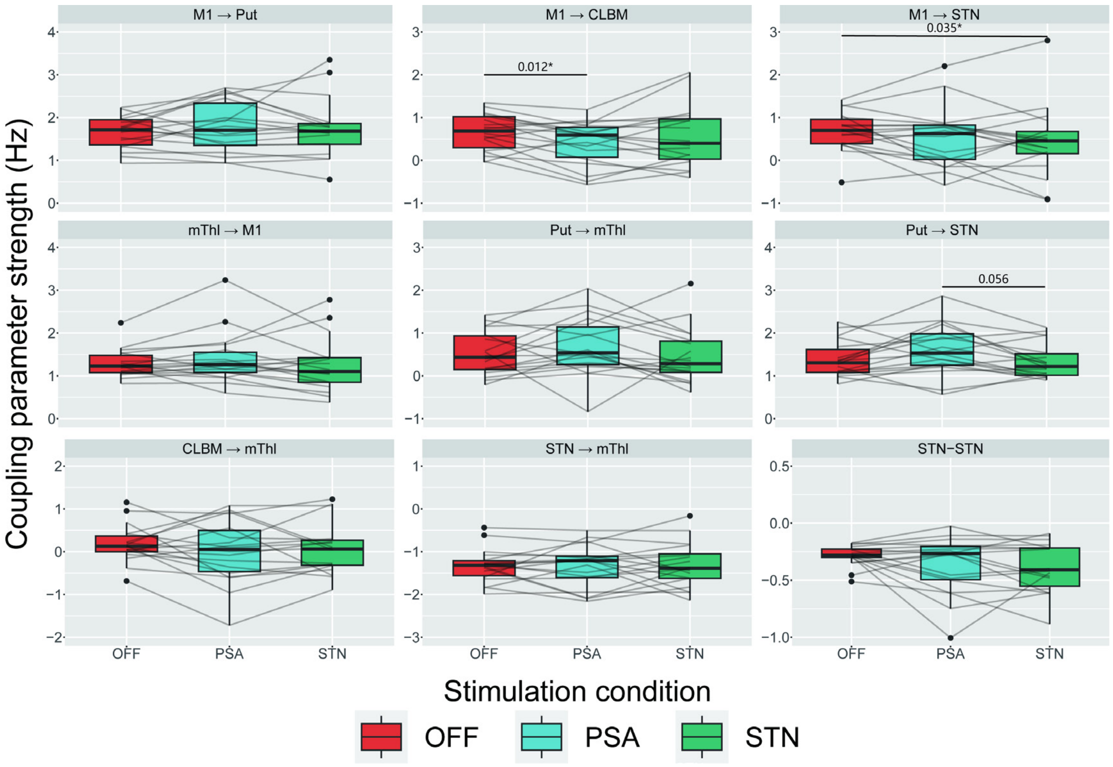

Supplementary Figure 2. The posterior estimate of coupling parameters within cortico-basal ganglia and cerebello-thalamo-cortical networks.

* Two upper outliers in the M1→STN panel were excluded from the statistical analysis.

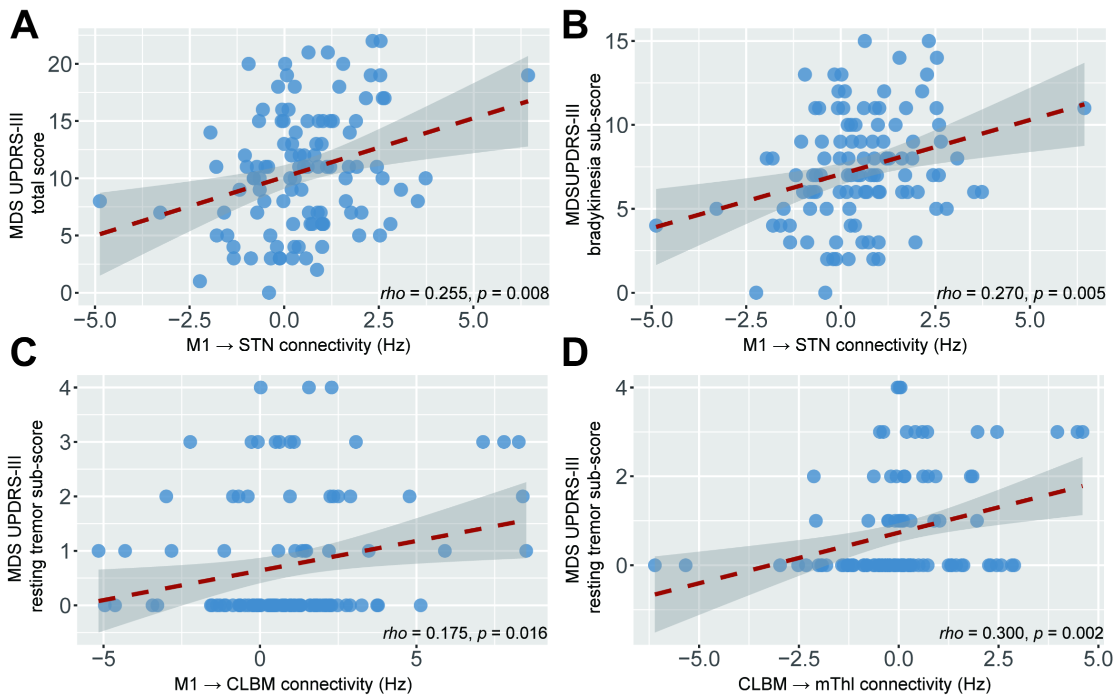

Supplementary Figure 3. Correlation between coupling parameters and motor performance across hemisphere and stimulation conditions. M1→STN connectivity strength was positively associated with total score (A) and bradykinesia sub-score (B) of the MDS UPDRS-III. The MDS UPDRS-III resting tremor sub-score was positively correlated with M1→CLBM (C) and CLBM→mThl (D) connectivity strength.
